# Association Between Benign Ovarian Tumors and Ovarian Cancer Risk: A Meta-Analysis of Ten Epidemiological Studies

**DOI:** 10.3389/fonc.2022.895618

**Published:** 2022-05-12

**Authors:** Jiao Guo, Haoshen Feng, Xi Gu

**Affiliations:** ^1^ Department of Oncology, Shengjing Hospital of China Medical University, Shenyang, China; ^2^ Department of Pulmonary and Critical Care Medicine, Shengjing Hospital of China Medical University, Shenyang, China

**Keywords:** benign ovarian tumors, epidemiological study, meta-analysis, ovarian cancer, incidence risk

## Abstract

**Background:**

Epidemiological evidence on the relationship between benign ovarian tumors and ovarian cancer risk has been controversial; therefore, this systematic review and meta-analysis evaluated this association.

**Methods:**

The PubMed and Web of Knowledge databases were searched for eligible studies published up to April 30, 2020. The study-specific risk estimates were pooled using a random-effects model.

**Results:**

Ten articles (two cohorts, seven case-control studies, and one pooled analysis of eight case-control studies) with 10331 ovarian cancer patients were included. Benign ovarian tumors were associated with an increased risk of ovarian cancer (pooled relative risk [RR]=1.39, 95% confidence interval [CI]: 1.01–1.90), with high heterogeneity among studies. The pooled RR was 2.02 (95%CI: 1.32–3.11) for two cohort studies, which was higher than the pooled result of eight case-control studies (pooled RR: 1.15; 95%CI: 0.92–1.44). When stratifying by histological type, the pooled RRs were 1.53 (95% CI: 0.37–6.29) and 3.62 (95%CI: 0.81–16.20) for serous and mucinous tumors, respectively. The pooled RRs were 1.61 (95%CI: 0.65–3.95) and 1.54 (95%CI: 1.29–1.84) for the associations of ovarian cyst with invasive and borderline cancers, respectively.

**Conclusions:**

Benign ovarian tumors were associated with an increased risk of ovarian cancer. Due to the high heterogeneity among the studies and the risks of bias, more studies are warranted to confirm these findings.

## Introduction

Ovarian cancer is one of the most common gynecologic cancers and also has the highest mortality rate (1, 2). Due to the lack of specific symptoms in the early stage, most ovarian cancers are diagnosed at advanced stages, with a 5-year survival rate of 20–30% (3, 4). Therefore, it is important to identify risk factors and develop systematic prevention and early detection strategies to reduce the risk of ovarian cancer.

Benign ovarian tumors may be precursors and etiological risk factors for ovarian cancer. While numerous epidemiological studies have investigated the association between benign ovarian tumors and ovarian cancer risk, the results have been inconsistent (5–14). Two large cohort studies based on the Danish Cancer Register indicated that benign ovarian tumors may be associated with a long-term increased risk of both mucinous and borderline ovarian cancers (7, 8). A population-based case-control study in a Chinese population reported an odds ratio of 12.00 (95% confidence interval [CI]: 2.50–57.70) for ovarian cancer among women with a prior ovarian cyst (14). However, other studies did not observe a statistically significant association. A pooled analysis of eight case-control studies with 5207 cases and 7705 controls in multiple countries suggested that ovarian cysts may not be associated with the risk of ovarian cancer (9).

Since the relationship between benign ovarian tumors and ovarian cancer risk remains unclear, this systematic review and meta-analysis aimed to assess the association based on the most recent epidemiological evidence.

## Materials and Methods

Because this was a study-level systematic review and meta-analysis of observational studies and did not involve the collection and analysis of any individual-level data, ethical approval was not sought for this study. This systematic review was conducted using the Preferred Reporting Items for Systematic Reviews and Meta-Analyses guidelines (15) (**Supplementary Table S1**).

### Literature Search and Study Selection

A comprehensive literature search of the PubMed and Web of Knowledge databases for publications indexed through April 2020 was performed using the following keywords: ((benign gynecologic conditions) OR (benign ovarian tumors) OR (ovarian cyst) OR (benign cyst)) AND ((ovarian) OR (ovary)) AND ((cancer) OR (neoplasm) OR (carcinoma) OR (tumor)). We also manually searched the reference lists of the relevant publications to identify additional studies. The inclusion criteria for the eligible studies were (1): observational studies (cohort, case-control, or cross-sectional) (2); studies that reported benign ovarian tumors as the exposure of interest and ovarian cancer risk as the outcome of interest; and (3) studies that provided useful risk estimates with 95% CIs, standard errors, or necessary data to calculate them. The exclusion criteria were (1): animal studies, letters, comments, conference abstracts, and grey literature (2); studies that did not report useful data; and (3) studies that were not published in English.

### Data Extraction

We used a pre-specified reporting form to extract the following data from each publication: first author, year of publication, study location, study design, sample size, exposure and assessment method, outcome and assessment methods, risk estimates with 95% CIs, and covariates included for adjustment in multivariable models. If multiple estimates were provided, priority was given to the multivariable-adjusted risk estimates, which were adjusted for the most potential confounding factors.

### Study Quality Assessment

Two authors independently evaluated study quality using a nine-point system based on the Newcastle-Ottawa quality assessment scale (16). The cohort studies were assessed based on population selection, group comparability, and outcome assessment. Case-control studies were assessed based on the selection of cases and controls, comparability of cases and controls, and exposure ascertainment.

### Statistical Analysis

We used relative risk (RR) as a measure of the association between benign ovarian tumors and ovarian cancer risk. The risk estimates were summarized using a random-effects model when significant heterogeneity was observed (17). Heterogeneity among studies was assessed using the *I* ^2^ statistic, with *I* ^2^ >50% indicating a heterogeneous result (18). We conducted subgroup analyses stratified by study design, geographic location, exposure assessment method, histological subtype, and invasiveness. We also performed a sensitivity analysis of the influence of individual studies on the summary estimate by repeating the meta-analysis after excluding one study at a time.

Publication bias was assessed by funnel plot and Begg’s and Egger’s tests (19, 20). When significant publication bias was detected, the trim-and-fill method was used to adjust for bias (21). The statistical analyses were conducted using Stata version 14.0 (StataCorp LP, College Station, Texas).

## Results

### Study Characteristics

**Figure 1:**
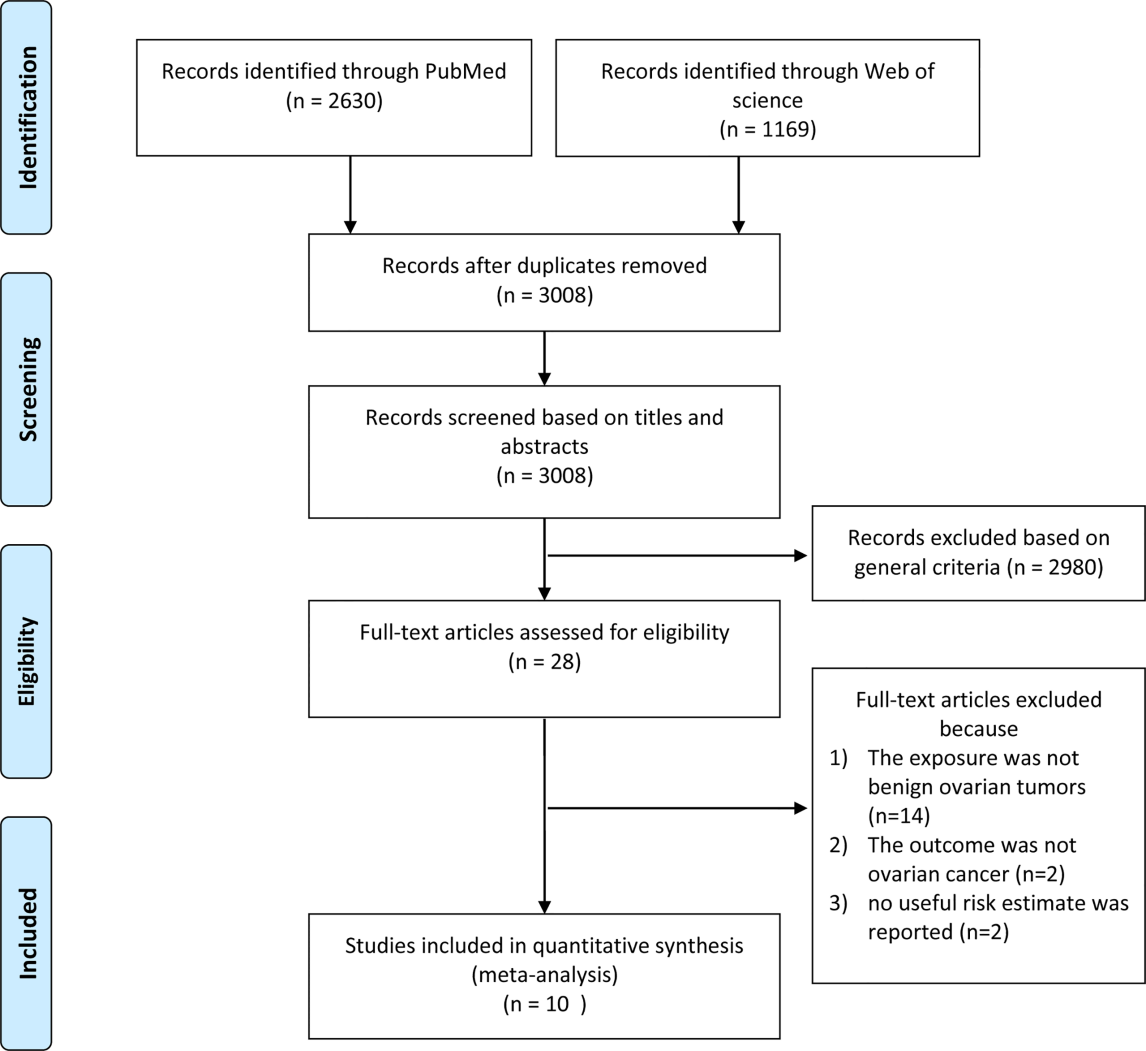
Flow diagram of study selection.

Our systematic literature search yielded 3799 records, the flow diagram of which is presented in **Figure 1**. After removing duplicates and screening the titles and abstracts, 28 articles were assessed for eligibility by full-text review. After a full-text review, ten studies (5–14) were finally included in this meta-analysis (**Table 1**).

**Table 1:** Characteristics of studies included in the meta-analysis.

Study	Location	Study design	No. of cases	No. of controls/size of cohort	Exposure & assessment	Outcome & assessment	Risk estimates (95% confidence interval)	Confounders adjustments
Guleria et al. (8)	Denmark	Cohort study	507	158221	Benign ovarian tumors; Solid tumors, Cystic tumors; & Clinically diagnosed	Ovarian cancer Serous/Mucinous/Endometrioid/Clear-cell & Clinically diagnosed	All benign tumors: 2.51 (2.38–2.64); Solid tumors: 2.07 (1.92–2.24); Cystic tumors: 3.10 (2.88–3.34)	N/A
Guleria et al. (7)	Denmark	Cohort study	216	139466	Benign ovarian tumors; Solid tumors; Cystic tumors; & Clinically diagnosed	Borderline ovarian tumor Serous/Mucinous & Clinically diagnosed	All benign ovarian tumors: 1.62 (1.43–1.82); Solid tumors: 1.78 (1.50–2.09); Cystic tumors: 1.46 (1.21–1.75)	N/A
Park et al. (12)	USA	Case–control study	600	752	Ovarian cyst & Self-reported	Ovarian cancer & Clinically diagnosed	Overall: 1.18 (0.83-1.69); Serous: 1.16 (0.76-1.75) Non-serous: 1.13 (0.68-1.90)	Age, study site, marital status, education, body mass index, parity, tubal ligation, duration of oral contraceptive use, family history of breast or ovarian cancer, talc use, endometriosis, fibroid, pelvic inflammatory disease
Rossing et al. (13)	USA	Case–control study	812	1313	Ovarian cyst & Self-reported	Ovarian cancer Borderline/Invasive Serous/Mucinous/Endometrioid/Clear-cell & Clinically diagnosed	All tumors: 1.1 (0.8, 1.3) Invasive tumors: 1.0 (0.7-1.3) Borderline tumors: 1.3 (0.9, 1.8)	Age, year of diagnosis/reference date, county of residence, number of full term births, duration of hormonal contraception
Borgfeldt et al. (5)	Sweden	Case–control study	N/A	N/A	Ovarian cysts Functional ovarian cysts & Clinically diagnosed	Ovarian cancer & Clinically diagnosed	Ovarian cyst: 0.86 (0.67-1.10); Functional cyst: 1.24 (0.81-1.89)	N/A
Ness et al. (9)	USA, Denmark, Australia, Canada	Case–control study	5207	7705	Ovarian cysts & Self-reported	Ovarian cancer & Clinically diagnosed	1.92 (0.83-4.46)	Age, gravidity, race, education, history of ovarian cancer, tubal ligation, duration of oral contraceptive use, research site, and different infertility type
Dal Maso et al. (6)	Italy	Case–control study	1031	2311	Ovarian cysts & Self-reported	Ovarian cancer Serous/Mucinous/Endometrioid & Clinically diagnosed	1.3 (0.9-1.8)	Age, parity, oral contraceptive use
Ness et al. (10)	USA	Case–control study	758	1362	Ovarian cysts & Self-reported	Ovarian cancer & Clinically diagnosed	1.3 (1.1-1.7)	Age, number of pregnancies, race, family history of ovarian cancer, oral contraceptive use, tubal ligation, hysterectomy, breast-feeding
Parazzini et al. (11)	Italy	Case–control study	971	2758	Ovarian cysts & Clinically diagnosed	Ovarian cancer & Clinically diagnosed	0.69 (0.41-1.13)	Age, education, parity, menopausal status, diabetes, thyroid disease, severe overweight, hypertension, cholelithiasis, hyperlipidaemia, benign female conditions, uterine leiomyomas, benign breast disease, previous breast biopsies
Shu et al. (14)	China	Case–control study	229	229	Ovarian cysts & Self-reported	Ovarian cancer & Clinically diagnosed	12.0 (2.5-57.7)	Age, education, number of livebirths, age at menarche

ICD, International Classification of Diseases; N/A, not available.

Two studies were cohort studies (7, 8) and eight were case-control studies (5, 6, 9–14). Ness et al. (9) used pooled data from eight case-control studies conducted between 1989 and 1999 (9). Five studies were conducted in Europe, three in North America, one in Asia, and one in multiple countries (the United States, Denmark, Canada, and Australia). To assess the exposure of benign ovarian tumors, six studies collected this information using self-reported approaches, while four studies collected this information by clinical diagnosis. Two studies reported results on serous tumors and three studies reported results on mucinous tumors. All studies scored more than seven stars in the study quality assessments, suggesting relatively high study quality (**Supplementary Tables S2, S3**).

### Associations Between Benign Ovarian Tumors and Ovarian Cancer Risk

**Figure 2:**
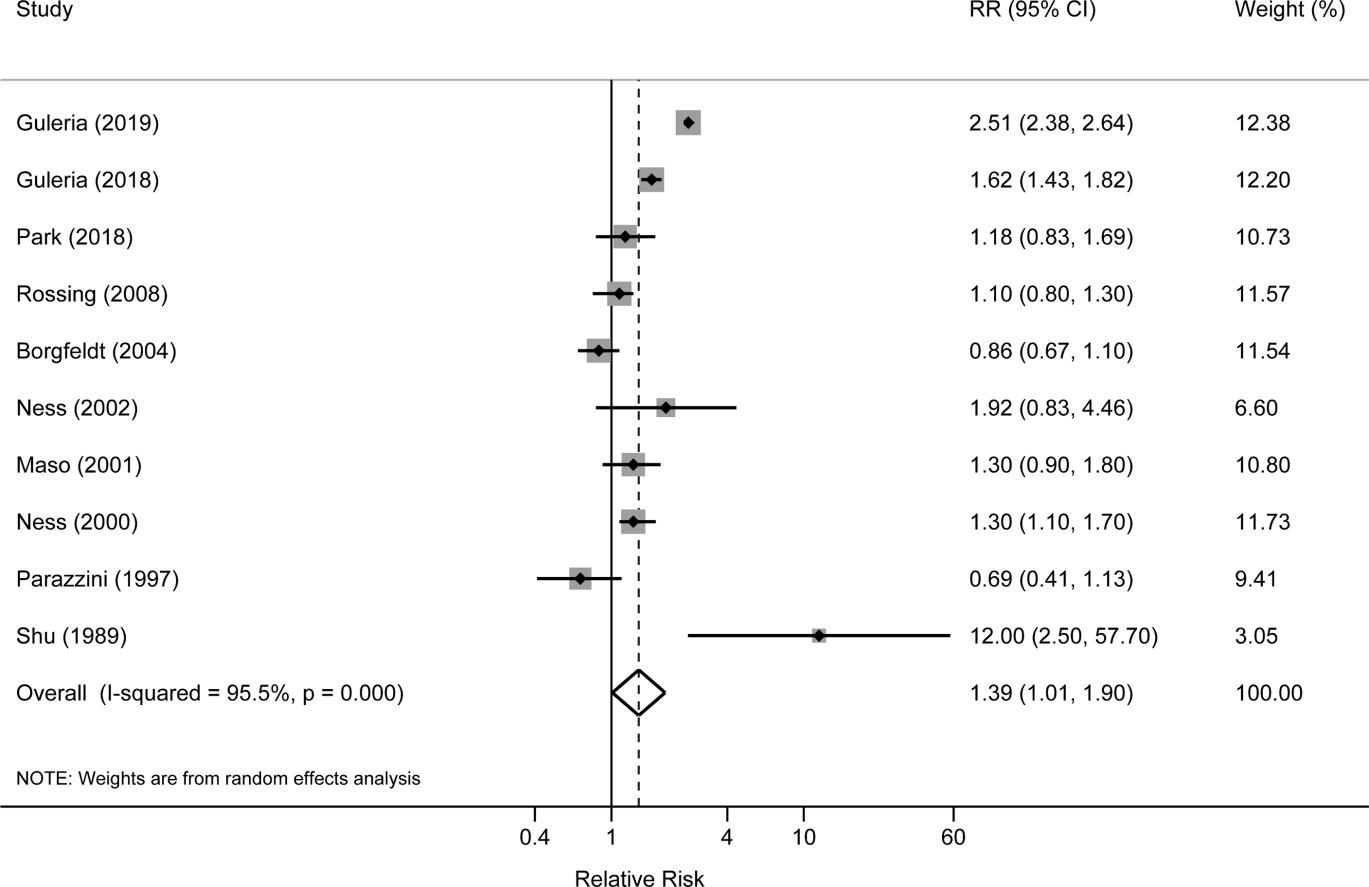
Forest plot for the association between benign ovarian tumors and risk of ovarian cancer.

The risk estimates for the association of benign ovarian tumors with the risk of ovarian cancer varied from 0.69 (95%CI: 0.41–1.13) in Parazzini et al. (11) to 12.00 (95%CI: 2.50–57.70) in Shu et al. (1989) (14) study. Four studies reported significantly increased risks of ovarian cancer associated with benign ovarian tumors; however, other studies did not observe a statistically significant association. After pooling the risk estimates from the 10 studies, benign ovarian tumors were associated with an increased risk of ovarian cancer (pooled RR=1.39, 95%CI: 1.01–1.90), with high heterogeneity among studies (*I* ^2 =^ 95.5%) (**Figure 2**). In the sensitivity analyses, we recalculated the pooled RRs by sequentially excluding one study. The study-specific RRs ranged from 1.23 (95% CI: 0.98–1.54) to 1.49 (95% CI: 1.08–2.05) after omission of the studies by Park et al. (12) and Dal Masso et al. (6), respectively.

In subgroup analysis stratified by study design, the pooled RR was 2.02 (95%CI: 1.32-3.11) for two cohort studies, which was higher than the pooled result of eight case-control studies (pooled RR: 1.15; 95%CI: 0.92–1.44). When stratifying by histological type, the pooled RRs were 1.53 (95% CI: 0.37–6.29) and 3.62 (95%CI: 0.81–16.20) for serous and mucinous tumors, respectively. The pooled RRs were 1.61 (95%CI: 0.65–3.95) and 1.54 (95%CI: 1.29–1.84) for the associations of ovarian cyst with invasive and borderline cancers, respectively. When stratified by study location, the pooled RRs were 1.20 (95%CI: 1.04–1.39) and 1.30 (95%CI: 0.86–1.98) in North America and European countries. One small case-control study conducted in Asia reported an RR of 12.00 (95%CI: 1.04–1.63) (**Table 2**).

**Table 2:** Associations of ovarian cysts with ovarian cancer risk stratified by subgroups.

Characteristics	No. of studies	RR (95%CI)	*I* ^2^ (%)	*P* value ^*^
**Study design**				0.072
Cohort study	2	2.02 (1.32-3.11)	97.7	
Case-control study	8	1.15 (0.92-1.44)	66.4	
**Geographic location**				0.369
North America	3	1.20 (1.04-1.39)	0.0	
Europe	5	1.30 (0.86-1.98)	97.0	
Asia	1	12.00 (2.50-57.70)	N/A	
**Exposure assessment**				0.778
Clinically diagnosed	4	1.30 (0.81-2.08)	97.6	
Self-reported	6	1.30 (1.04-1.63)	51.5	
**Histological types**				0.803
Serous	4	1.53 (0.37-6.29)	99.7	
Mucinous	3	3.62 (0.81-16.20)	99.4	
Others	3	1.05 (0.72-1.53)	64.4	
**Invasiveness**				0.845
Invasive	2	1.61 (0.65-3.95)	97.0	
Borderline	2	1.54 (1.29-1.84)	27.6	

CI, confidence interval; N/A, not available; RR, relative risk.

^*^ P value for heterogeneity between groups.

Publication bias was possible (Egger’s test: *P*=0.026; Begg’s test: *P*=0.325). (**Supplementary Figure S1**). The results after applying the trim-and-fill method to adjust for bias showed a pooled RR consistent with the main result.

## Discussion

This is the first systematic review and meta-analysis to summarize the epidemiological evidence of the association between benign ovarian tumors and the risk of ovarian cancer. In this study, a 39% increased risk of ovarian cancer was associated with a history of benign ovarian tumors. The direction of the association was consistent in most subgroups and the results were robust in the sensitivity analysis.

The biological mechanisms by which benign ovarian tumors may transform into malignant tumors are unknown; however, the area at which the epithelium transitions from the ovarian to tubal may be highly susceptible to neoplastic changes (22). The results of animal studies have indicated that the ovarian surface epithelium is the precursor tissue for ovarian neoplasms, which are more susceptible to malignant transformation upon exposure to certain stimulants (23). Furthermore, genetic risk factors such as KRAS and p53 mutations may play important roles in the transition from benign ovarian tumors to ovarian cancer (24, 25).

The results of the present study indicated that the associations may vary by histological type, with a higher risk of mucinous ovarian cancer. Consistent with our results, a nationwide cohort study reported that women with benign ovarian tumors were at an increased risk of mucinous ovarian cancer, with both solid and cystic benign ovarian tumors associated with an increased risk (8). Women with benign ovarian tumors have increased risks for subsequent borderline ovarian tumors, including both serous and mucinous histological types of ovarian tumors (7). In a small case-control study of 215 women with borderline ovarian tumors and 1313 control women, Rossing et al. reported that women with ovarian cysts were at a higher risk of borderline mucinous tumors but not borderline serous tumors (13). Thus, our findings add to the knowledge that different histological types of ovarian cancer might have varied etiologies (26) and suggest that benign ovarian tumors might be precursors of mucinous ovarian cancer. The underlying reasons for such a specific association may be partly explained by a shared environmental and genetic etiology between benign ovarian tumors and mucinous ovarian cancer (27). For example, tobacco smoking and obesity are shared risk factors for both benign ovarian tumors, specifically mucinous ovarian cancer (13, 28, 29). In contrast, current evidence fails to identify common risk factors for both benign ovarian tumors and any of the other histological types of ovarian cancer (30, 31). Furthermore, some genetic mutations, such as KRAS mutations, are more likely to occur in mucinous benign ovarian tumors and mucinous ovarian cancers (32). Taken together, compared to other histological types of ovarian cancer, mucinous ovarian cancer may be more likely to have a shared etiology with benign ovarian tumors, which may explain the findings of the present study.

Our study has several strengths. First, to our knowledge, this is the first systematic review meta-analysis to comprehensively summarize the association between benign ovarian tumors and ovarian cancer risk. Second, our study had a large sample size of 10,331 ovarian patients, which provided sufficient statistical power to detect even a modest association. Third, the histological types of ovarian cancer were considered when evaluating the association between benign ovarian tumors and ovarian cancer. Finally, our study has important clinical implications by suggesting that benign ovarian tumors might be precursors to mucinous ovarian cancer and providing a theoretical basis for clinical decision-making and ovarian cancer prevention and treatment. However, this study has some limitations. First, most of the included studies had case-control designs, which increases the risk for selection and recall biases (33), especially for patients whose exposure status was collected based on self-reports. Nevertheless, the results were similar between the self-reported and clinical diagnosis/ascertainment subgroups in the subgroup analysis stratified by exposure assessment methods. Second, residual confounding may distort the association between benign ovarian tumors and ovarian cancer; we were unable to address these problems due to confounding inherent in the original studies. Third, detection bias was possible as women with benign ovarian tumors tend to undergo more regular and extensive physical examinations; thus, they are more likely to have an early diagnosis of ovarian cancer. Of note, a Danish cohort study, after excluding the first year of follow-up from the analyses (when the likelihood of detection bias was highest), reported that the increased risk for ovarian tumors persisted for up to 9 years compared to that of the general female population (7). Fourth, high heterogeneity was detected across the included studies. The heterogeneity may come from various sources, such as study design, study population, and exposure assessment methods. Subgroup analysis and meta-regression to explore the potential sources of heterogeneity showed a tendency for slight attenuation of the heterogeneity in several subgroup analyses. Finally, the possibility of publication bias cannot be excluded. Although the overall results did not change materially after adjusting for the trim-and-fill method, the summary risk estimates should be interpreted with caution.

## Conclusions

The results of this study suggest that benign ovarian tumors are associated with an increased risk of developing ovarian cancer. Due to the high heterogeneity among the studies and the risk of bias, more studies are warranted to confirm our findings. As benign ovarian tumors and ovarian cancer share some environmental and genetic etiologies, benign ovarian tumors may be premalignant. Therefore, potential prevention and treatment initiatives, such as more accurate screening strategies and ovarian surgery after diagnosis, should be encouraged to reduce the future risk of ovarian cancer.

## Data Availability Statement

The original contributions presented in the study are included in the article/**Supplementary Material**. Further inquiries can be directed to the corresponding author.

## Author Contributions

JG and XG were involved in the conceptualization of the study. JG collected the data. HF and XG conducted the analysis. XG contributed to writing. All authors contributed to the article and approved the submitted version

## Conflict of Interest

The authors declare that the research was conducted in the absence of any commercial or financial relationships that could be construed as a potential conflict of interest.

## Publisher’s Note

All claims expressed in this article are solely those of the authors and do not necessarily represent those of their affiliated organizations, or those of the publisher, the editors and the reviewers. Any product that may be evaluated in this article, or claim that may be made by its manufacturer, is not guaranteed or endorsed by the publisher.

## References

[1] Momenimovahed Z Tiznobaik A Taheri S Salehiniya H . Ovarian Cancer in the World: Epidemiology and Risk Factors. Int J Womens Health (2019) 11:287–99. doi: 10.2147/IJWH.S197604 PMC6500433 31118829

[2] Reid BM Permuth JB Sellers TA . Epidemiology of Ovarian Cancer: A Review. Cancer Biol Med (2017) 14(1):9–32. doi: 10.20892/j.issn.2095-3941.2016.0084 28443200 PMC5365187

[3] Edwards HM Noer MC Sperling CD Nguyen-Nielsen M Lundvall L Christensen IJ . Survival of Ovarian Cancer Patients in Denmark: Results From the Danish Gynaecological Cancer Group (DGCG) Database, 1995-2012. Acta Oncol (2016) 55 Suppl 2:36–43. doi: 10.1080/0284186X.2016.1182641 27355258

[4] Torre LA Trabert B DeSantis CE Miller KD Samimi G Runowicz CD . Ovarian Cancer Statistics, 2018. CA Cancer J Clin (2018) 68(4):284–96. doi: 10.3322/caac.21456 PMC6621554 29809280

[5] Borgfeldt C Andolf E . Cancer Risk After Hospital Discharge Diagnosis of Benign Ovarian Cysts and Endometriosis. Acta Obstet Gynecol Scand (2004) 83(4):395–400. doi: 10.1111/j.0001-6349.2004.00305.x 15005789

[6] Dal Maso L Canzonieri V Talamini R Franceschi S La Vecchia C . Origin of Ovarian Cancer From Benign Cysts. Eur J Cancer Prev (2001) 10(2):197–9. doi: 10.1097/00008469-200104000-00016 11330466

[7] Guleria S Jensen A Kjaer SK . Risk of Borderline Ovarian Tumors Among Women With Benign Ovarian Tumors: A Cohort Study. Gynecol Oncol (2018) 148(1):86–90. doi: 10.1016/j.ygyno.2017.11.024 29174556

[8] Guleria S Jensen A Toender A Kjaer SK . Risk of Epithelial Ovarian Cancer Among Women With Benign Ovarian Tumors: A Follow-Up Study. Cancer Causes Control (2020) 31(1):25–31. doi: 10.1007/s10552-019-01245-4 31673820

[9] Ness RB Cramer DW Goodman MT Kjaer SK Mallin K Mosgaard BJ . Infertility, Fertility Drugs, and Ovarian Cancer: A Pooled Analysis of Case-Control Studies. Am J Epidemiol (2002) 155(3):217–24. doi: 10.1093/aje/155.3.217 11821246

[10] Ness RB Grisso JA Cottreau C Klapper J Vergona R Wheeler JE . Factors Related to Inflammation of the Ovarian Epithelium and Risk of Ovarian Cancer. Epidemiology (2000) 11(2):111–7. doi: 10.1097/00001648-200003000-00006 11021606

[11] Parazzini F Moroni S La Vecchia C Negri E dal Pino D Bolis G . Ovarian Cancer Risk and History of Selected Medical Conditions Linked With Female Hormones. Eur J Cancer (1997) 33(10):1634–7. doi: 10.1016/s0959-8049(97)00011-7 9389926

[12] Park HK Schildkraut JM Alberg AJ Bandera EV Barnholtz-Sloan JS Bondy M . Benign Gynecologic Conditions are Associated With Ovarian Cancer Risk in African-American Women: A Case-Control Study. Cancer Causes Control (2018) 29(11):1081–91. doi: 10.1007/s10552-018-1082-4 PMC6230481 30269307

[13] Rossing MA Cushing-Haugen KL Wicklund KG Doherty JA Weiss NS . Risk of Epithelial Ovarian Cancer in Relation to Benign Ovarian Conditions and Ovarian Surgery. Cancer Causes Control (2008) 19(10):1357–64. doi: 10.1007/s10552-008-9207-9 PMC2751585 18704718

[14] Shu XO Brinton LA Gao YT Yuan JM . Population-Based Case-Control Study of Ovarian Cancer in Shanghai. Cancer Res (1989) 49(13):3670–4. 2731180

[15] Moher D Liberati A Tetzlaff J Altman DG Group P . Preferred Reporting Items for Systematic Reviews and Meta-Analyses: The PRISMA Statement. PloS Med (2009) 6(7):e1000097. doi: 10.1371/journal.pmed.1000097 19621072 PMC2707599

[16] Wells GA SB O’Connell D Peterson J Welch V Losos M . The Newcastle-Ottawa Scale (NOS) for Assessing the Quality If Nonrandomized Studies in Meta-Analyses. Available at: http://www.ohri.ca/programs/clinical_epidemiology/oxford.htm.

[17] DerSimonian R Laird N . Meta-Analysis in Clinical Trials Revisited. Contemp Clin Trials (2015) 45(Pt A):139–45. doi: 10.1016/j.cct.2015.09.002 PMC4639420 26343745

[18] Higgins JP Thompson SG . Quantifying Heterogeneity in a Meta-Analysis. Stat Med (2002) 21(11):1539–58. doi: 10.1002/sim.1186 12111919

[19] Begg CB Mazumdar M . Operating Characteristics of a Rank Correlation Test for Publication Bias. Biometrics (1994) 50(4):1088–101. doi: 10.2307/2533446 7786990

[20] Egger M Davey Smith G Schneider M Minder C . Bias in Meta-Analysis Detected by a Simple, Graphical Test. BMJ (1997) 315(7109):629–34. doi: 10.1136/bmj.315.7109.629 PMC2127453 9310563

[21] Duval S Tweedie R . Trim and Fill: A Simple Funnel-Plot-Based Method of Testing and Adjusting for Publication Bias in Meta-Analysis. Biometrics (2000) 56(2):455–63. doi: 10.1111/j.0006-341x.2000.00455.x 10877304

[22] Auersperg N Woo MM Gilks CB . The Origin of Ovarian Carcinomas: A Developmental View. Gynecol Oncol (2008) 110(3):452–4. doi: 10.1016/j.ygyno.2008.05.031 18603285

[23] Orsulic S Li Y Soslow RA Vitale-Cross LA Gutkind JS Varmus HE . Induction of Ovarian Cancer by Defined Multiple Genetic Changes in a Mouse Model System. Cancer Cell (2002) 1(1):53–62. doi: 10.1016/s1535-6108(01)00002-2 12086888 PMC2267863

[24] Garrett AP Lee KR Colitti CR Muto MG Berkowitz RS Mok SC . K-Ras Mutation May Be an Early Event in Mucinous Ovarian Tumorigenesis. Int J Gynecol Pathol (2001) 20(3):244–51. doi: 10.1097/00004347-200107000-00007 11444200

[25] Cheng EJ Kurman RJ Wang M Oldt R Wang BG Berman DM . Molecular Genetic Analysis of Ovarian Serous Cystadenomas. Lab Invest (2004) 84(6):778–84. doi: 10.1038/labinvest.3700103 15077125

[26] Wentzensen N Poole EM Trabert B White E Arslan AA Patel AV . Ovarian Cancer Risk Factors by Histologic Subtype: An Analysis From the Ovarian Cancer Cohort Consortium. J Clin Oncol (2016) 34(24):2888–98. doi: 10.1200/jco.2016.66.8178 PMC5012665 27325851

[27] Jordan SJ Green AC Whiteman DC Webb PM . Risk Factors for Benign, Borderline and Invasive Mucinous Ovarian Tumors: Epidemiological Evidence of a Neoplastic Continuum? Gynecol Oncol (2007) 107(2):223–30. doi: 10.1016/j.ygyno.2007.06.006 17662378

[28] Wyshak G Frisch RE Albright TE Albright NL Schiff I . Smoking and Cysts of the Ovary. Int J Fertil (1988) 33(6):398–404. 2906914

[29] Jordan SJ Green AC Whiteman DC Webb PM . Risk Factors for Benign Serous and Mucinous Epithelial Ovarian Tumors. Obstet Gynecol (2007) 109(3):647–54. doi: 10.1097/01.AOG.0000254159.75977.fa 17329516

[30] Rasmussen CB Kjaer SK Albieri V Bandera EV Doherty JA Hogdall E . Pelvic Inflammatory Disease and the Risk of Ovarian Cancer and Borderline Ovarian Tumors: A Pooled Analysis of 13 Case-Control Studies. Am J Epidemiol (2017) 185(1):8–20. doi: 10.1093/aje/kww161 27941069 PMC5209588

[31] Booth M Beral V Maconochie N Carpenter L Scott C . A Case-Control Study of Benign Ovarian Tumours. J Epidemiol Commun Health (1992) 46(5):528–31. doi: 10.1136/jech.46.5.528 PMC1059646 1479325

[32] Thomas NA Neville PJ Baxter SW Campbell IG . Genetic Analysis of Benign Ovarian Tumors. Int J Cancer (2003) 105(4):499–505. doi: 10.1002/ijc.11107 12712441

[33] Ba DM Ssentongo P Beelman RB Muscat J Gao X Richie JP . Higher Mushroom Consumption Is Associated With Lower Risk of Cancer: A Systematic Review and Meta-Analysis of Observational Studies. Adv Nutr (2021) 12(5):1691–704. doi: 10.1093/advances/nmab0155 PMC8483951 33724299

