# Supplementary figures and images for: Association Between Benign Ovarian Tumors and Ovarian Cancer Risk: A Meta-Analysis of Ten Epidemiological Studies
Source: Front Oncol. 2022 May 12;12:895618. doi: 10.3389/fonc.2022.895618 (PMC9133501; doi:10.3389/fonc.2022.895618)

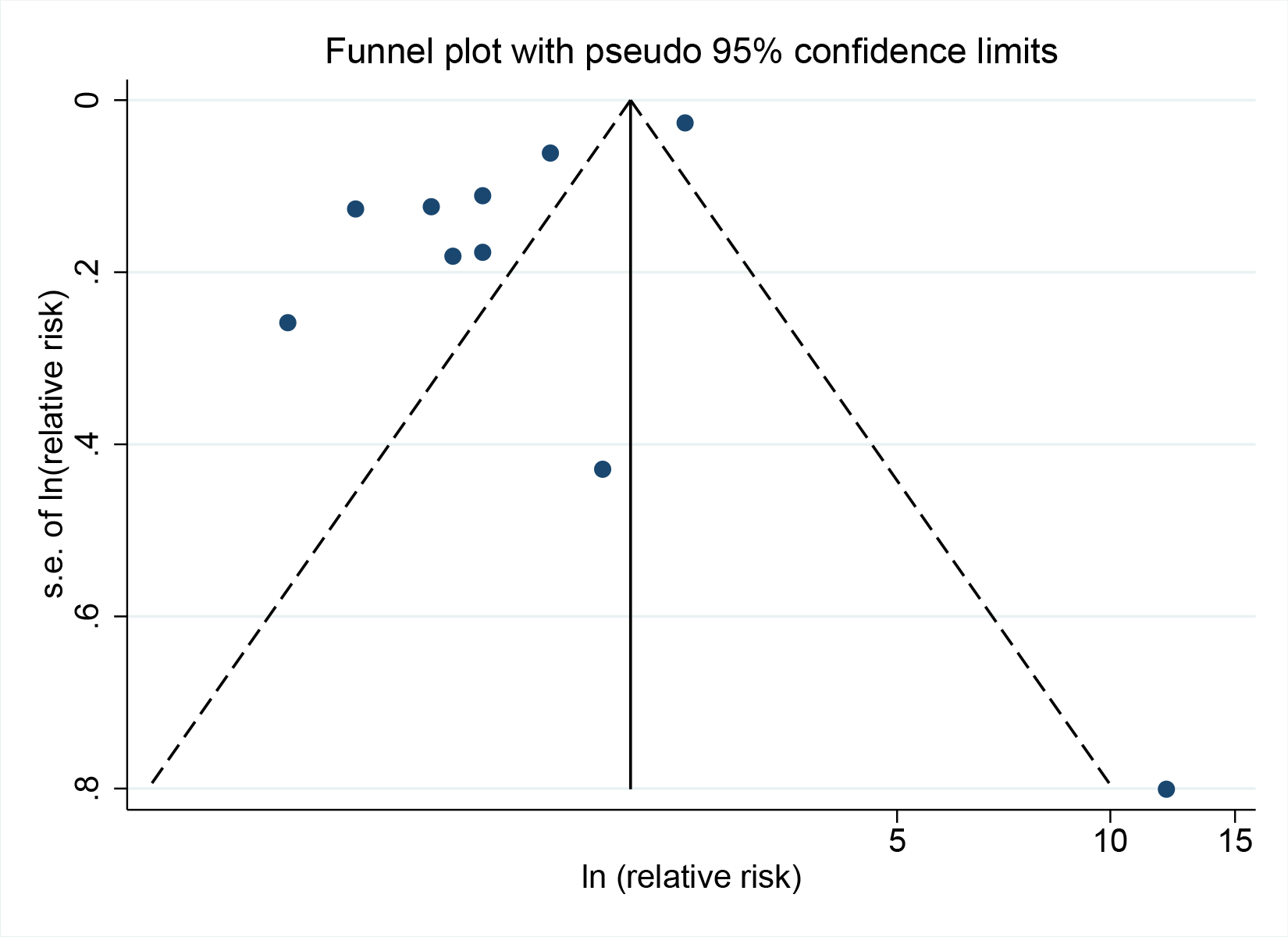

Supplement: Supplementary file 1 [file Image_1.tif]
