# Supplementary material for: Association Between Benign Ovarian Tumors and Ovarian Cancer Risk: A Meta-Analysis of Ten Epidemiological Studies
Source: Front Oncol. 2022 May 12;12:895618. doi: 10.3389/fonc.2022.895618 (PMC9133501; doi:10.3389/fonc.2022.895618)
Supplement: Supplementary file 3 [file Table_2.docx]

**Table S2. Study quality of cohort studies included in the meta-analysis**

| **Study** | **Selection** | | | | **Comparability** | **Outcome** | | |
| --- | --- | --- | --- | --- | --- | --- | --- | --- |
|  | **Representativeness of the exposed cohort** | **Selection of the unexposed**  **cohort** | **Ascertainment**  **of exposure** | **Outcome of interest not present at start of study** | **Control for**  **potential confounders†** | **Assessment of outcome** | **Follow-up**  **long enough for outcomes**  **to occur ‡** | **Adequacy of**  **follow-up**  **of cohorts §** |
| Guleria et al, 2020 | ⚝ | ⚝ | ⚝ | ⚝ | - | ⚝ | ⚝ | ⚝ |
| Guleria et al, 2018 | ⚝ | ⚝ | ⚝ | ⚝ | - | ⚝ | ⚝ | ⚝ |

† A maximum of 2 stars could be awarded for this item. Studies that controlled for age received one star, whereas studies that controlled for other important confounders received an additional star.

‡ A cohort study with a follow-up time >10 year was assigned one star.

§ A cohort study with a follow-up rate >75% was assigned one star.

**Table S3. Study quality of case-control studies included in the meta-analysis**

| **Study** | **Selection** | | | | **Comparability** | **Exposure** | | |
| --- | --- | --- | --- | --- | --- | --- | --- | --- |
|  | **Adequate definition of cases** | **Representativeness  of cases** | **Selection  of  control  subjects** | **Definition of control  subjects** | **Control for potential confounders†** | **Exposure evaluation** | **Same way of ascertainment  for all  subjects** | **Non-response rate‡** |
| Park et al, 2018 | ⚝ | ⚝ | ⚝ | ⚝ | ⚝⚝ | - | ⚝ | - |
| Rossing et al, 2008 | ⚝ | ⚝ | ⚝ | ⚝ | ⚝⚝ | - | ⚝ | ⚝ |
| Borgfeldt et al, 2004 | ⚝ | ⚝ | ⚝ | ⚝ | - | ⚝ | ⚝ | ⚝ |
| Ness et al, 2002 | ⚝ | ⚝ | ⚝ | ⚝ | ⚝⚝ | - | ⚝ | - |
| Dal Maso et al, 2001 | ⚝ | ⚝ | ⚝ | ⚝ | ⚝⚝ | - | ⚝ | - |
| Ness et al, 2000 | ⚝ | ⚝ | ⚝ | ⚝ | ⚝⚝ | - | ⚝ | ⚝ |
| Parazzini et al, 1997 | ⚝ | ⚝ | ⚝ | ⚝ | ⚝⚝ | ⚝ | ⚝ | - |
| Shu et al, 1989 | ⚝ | ⚝ | ⚝ | ⚝ | ⚝⚝ | - | ⚝ | ⚝ |

† A maximum of two stars could be awarded for this item. Studies that controlled for total energy intake received one star, whereas studies that controlled for other important confounders received an additional star.

‡ One star was assigned if there was no significant difference in the response rate between control subjects and cases by using the chi-square test (P>0.05)
